# Supplementary material for: Epigenome analysis of an algae-infecting giant virus reveals a unique methylation motif catalogue
Source: PLoS One. 2025 Dec 12;20(12):e0330887. doi: 10.1371/journal.pone.0330887 (PMC12700378; doi:10.1371/journal.pone.0330887)
Supplement: S1 File — (DOCX) [file pone.0330887.s001.docx]

**SUPPLEMENTAL ONLINE INFORMATION**

For publication in conjunction with the following:

**Epigenome analysis of an algae-infecting giant virus reveals a unique methylation motif catalogue**

Alexander R. Truchon^1^, Erik R. Zinser^1^, Steven W. Wilhelm^1*^

^1^Department of Microbiology, University of Tennessee, Knoxville, TN, USA

**^*^Corresponding author:** Steven W. Wilhelm, email: [wilhelm@utk.edu](mailto:wilhelm@utk.edu)

**Key words:** methyltransferase, *Nucleocytoviricota*, long-read sequencing, endonuclease, *Aureococcus anophagefferens,* N^6^-methyladenine, virocell, hemi-methylation


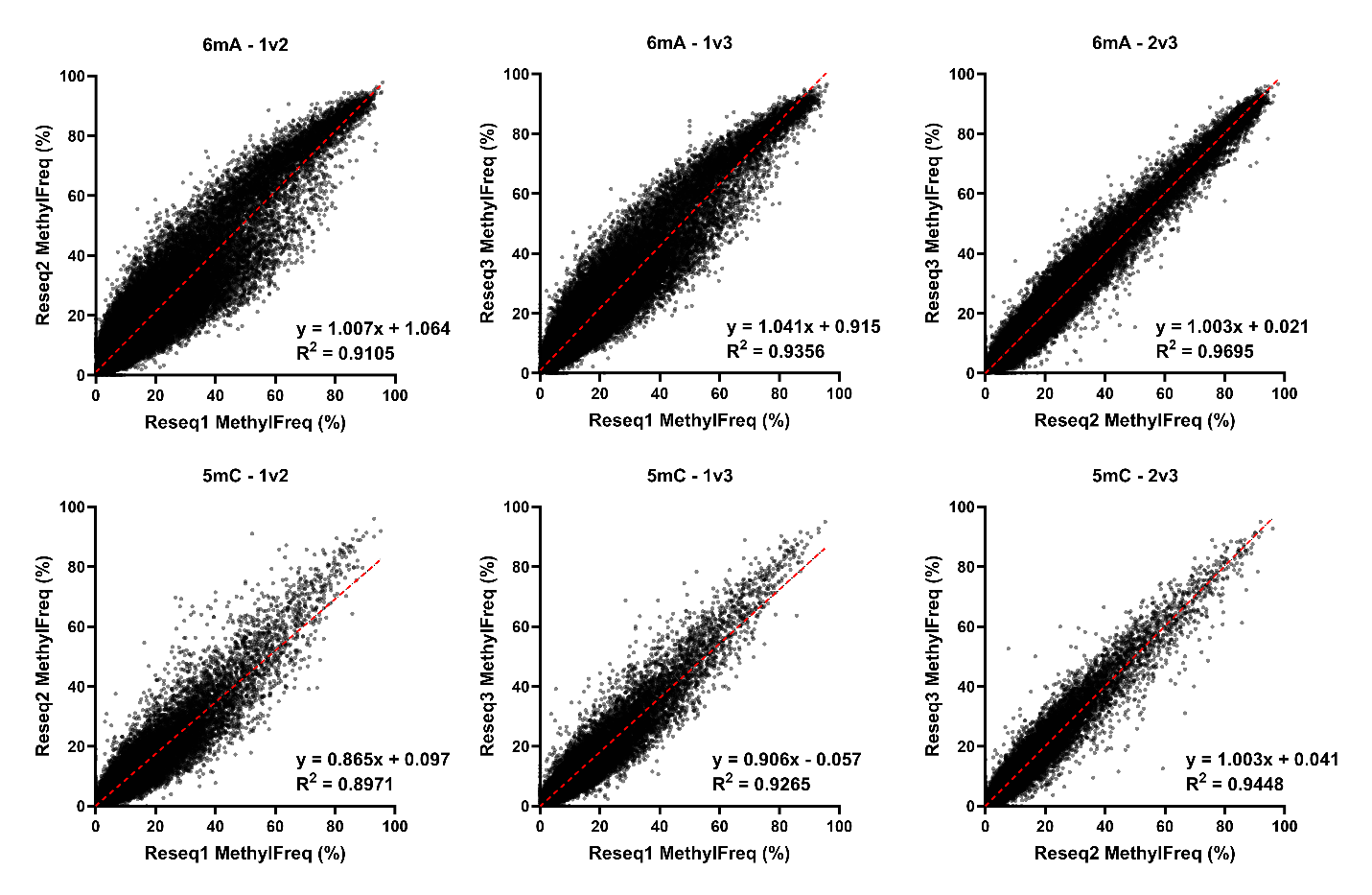


Supplemental Figure 1: Methylation frequency score of each adenine (top) or cytosine (bottom) in the AaV genome compared between distinct sequencing libraries. Red dotted lines portray simple linear regressions of the data.


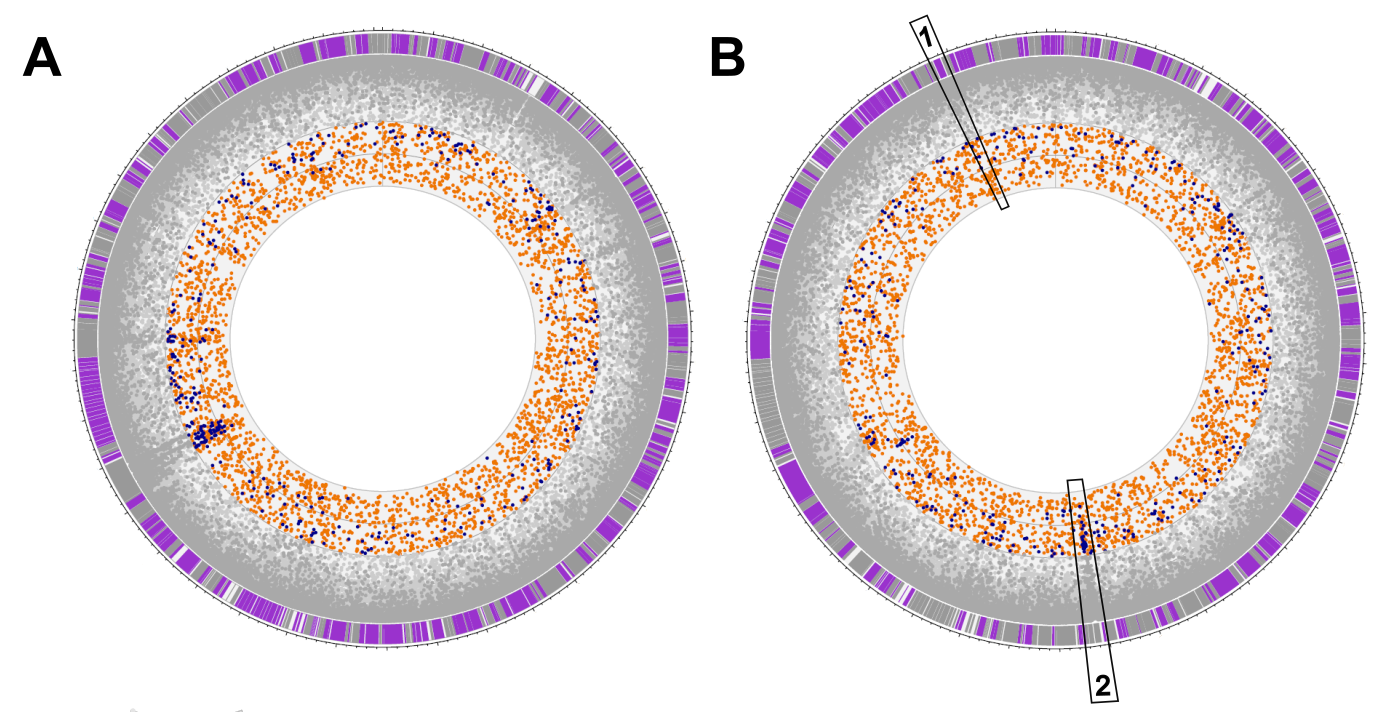


Supplemental Figure 2. Genomic maps of methylation frequency on the positive (A) and negative (B) strands of AaV, with the inner ring representing 100% methylation and the outer ring representing 0%. Sites below 50% methylaton frequency are shaded gray, cytosines above 50% are colored blue and adenines above 50% are colored orange. Genes on the sense strand for each map are purple while genes on the antisense strand are gray. Segments 1 and 2 of 2B are enhanced in 2A and 2B, respectively, representing the major capsid protein gene region and a highly repetitive region enriched in 5mC.


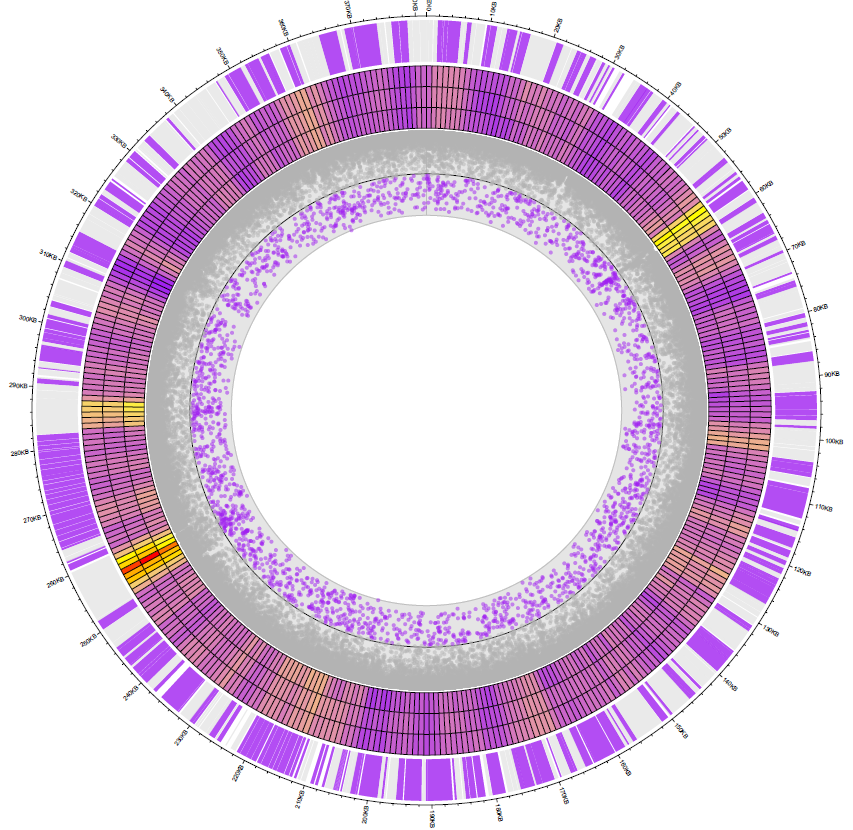

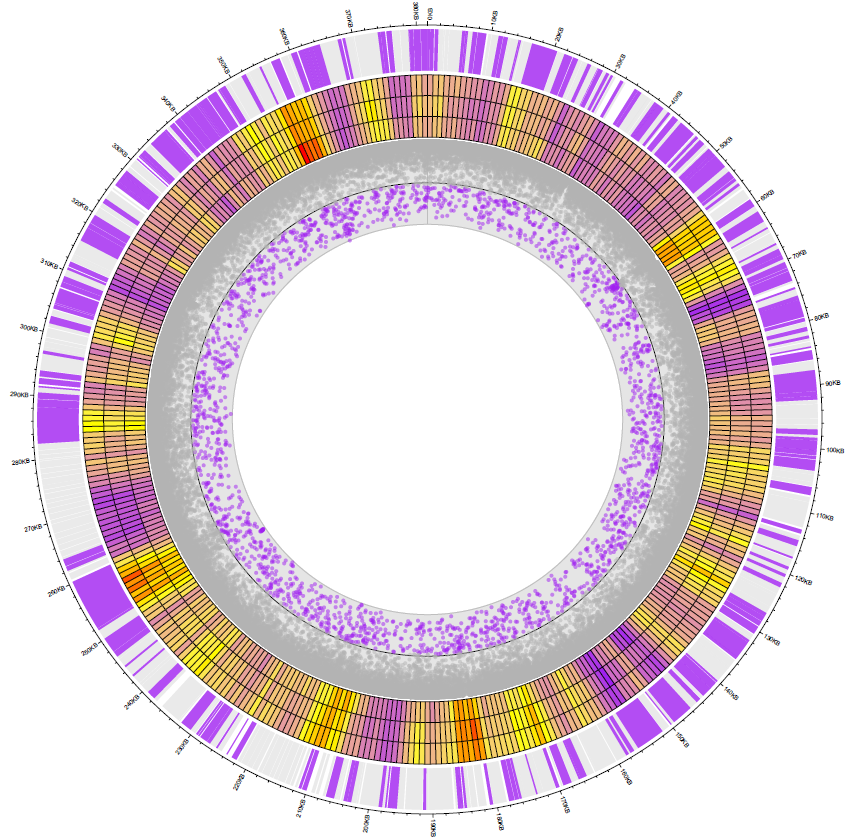


Supplemental Figure 3. Adenine methylation frequency and enrichment maps of AaV on the positive (left) and negative (right) strand. Outer ring: Genes on the coding strand are displayed in purple, while genes on the template strand are displayed in gray. Middle ring: Enrichment of methylation based on MethylFreq Z-score of 1000 bp sliding windows on the coding strand, red implicating a high enrichment and purple representing a low enrichment. Each ring of the heatmap represents calculated scores from different sequencing libraries. Inner ring: MethylFreq of each site with sites lower than 50% blotted out in gray.


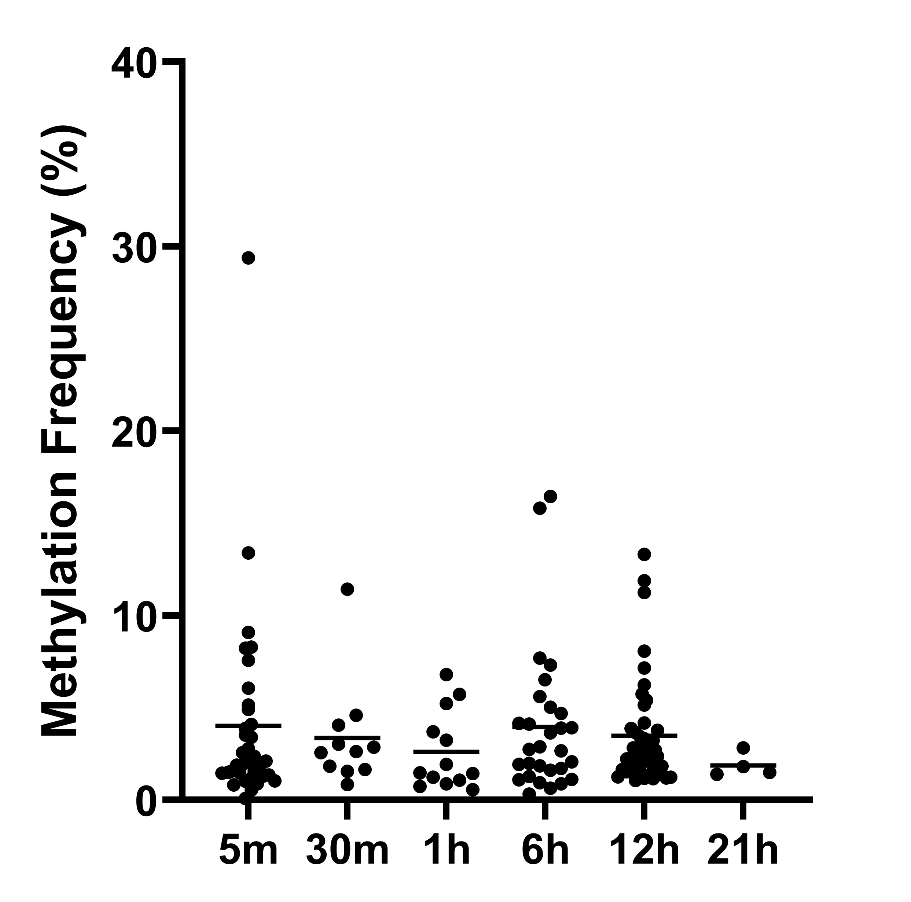


Supplemental Figure 4. Time of expression during infection of annotated genes in AaV and the average methylation frequency across the 50 bp region upstream of the gene’s start codon.


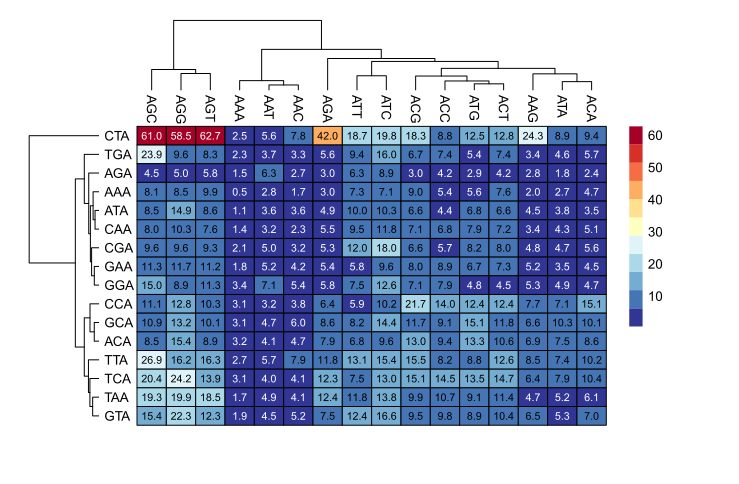


Supplemental Figure 5. Clustered heatmap displaying methylation frequencies of all potential adenine-centric pentamers, rows representing the two nucleotides upstream of the base in question and columns representing the two nucleotides downstream.


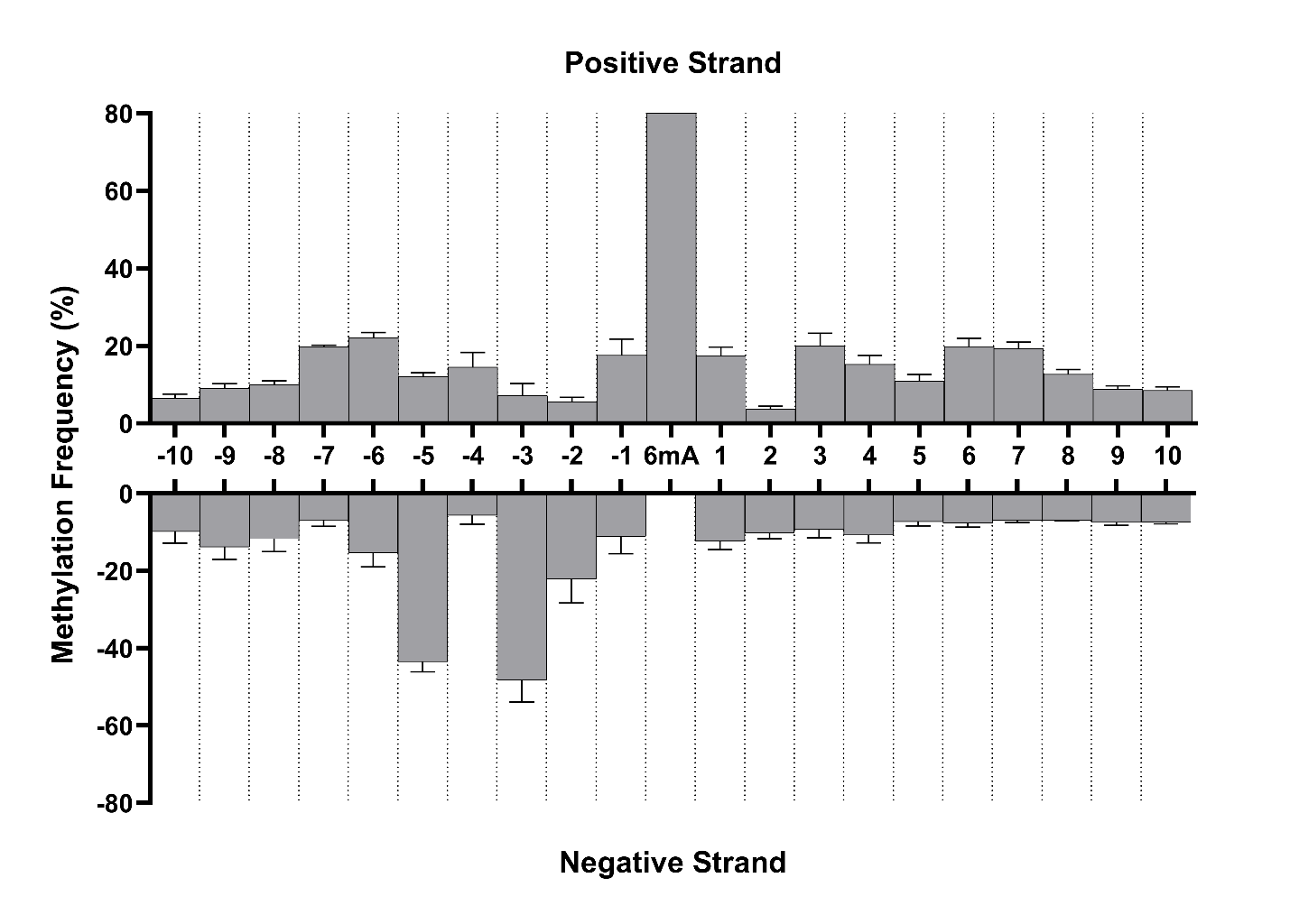


Supplemental Figure 6: Average methylation frequency of adenines surrounding 6mA adenines methylated at a frequency of at least 80% on the sense (top) and antisense (bottom) strand.


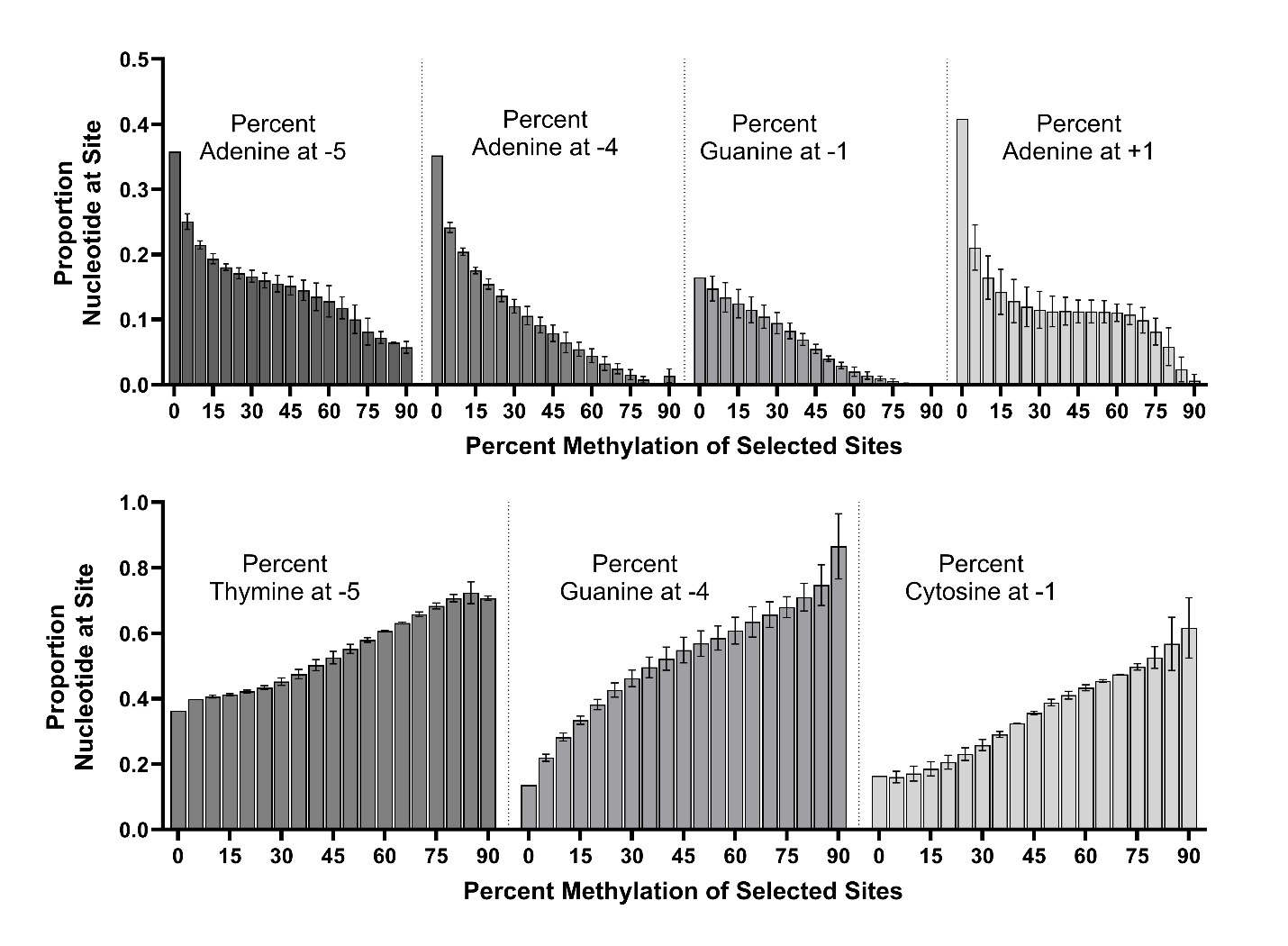


Supplemental Figure 7: Proportion of specific nucleotides at defined sites upstream or downstream of adenines as a function of the adenine’s methylation frequency. When only considering the highly methylated adenines, surrounding nucleotides shift from their expected genomic proportions (*i.e.* ~35% for adenines and thymines or 15% for guanines and cytosines).


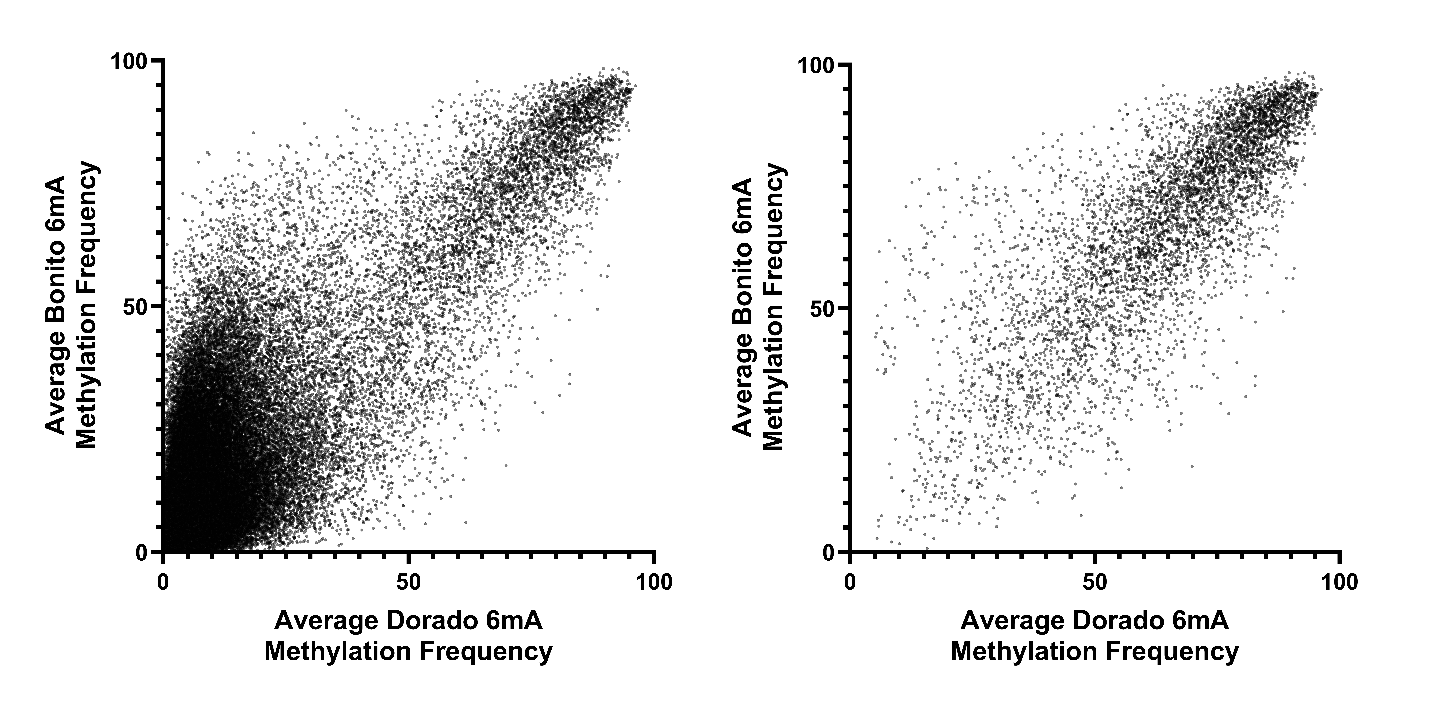


Supplemental Figure 8. Average methylation frequency for all AaV adenines (A) and AaV adenines that belong to one of the motifs described in Table 1. Methylation frequencies were generated using two basecallers, Nanopore dorado and Bonito. Sites with less than 30 AaV reads mapped were not included.


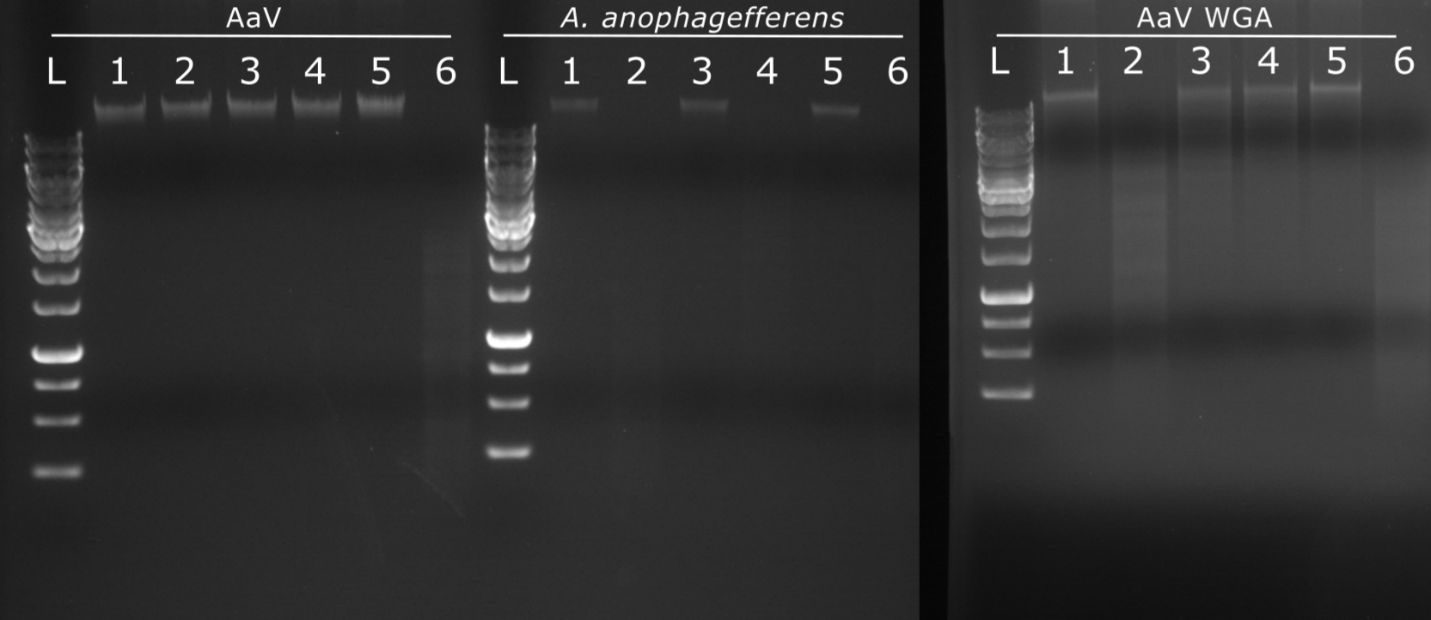


Supplemental Figure 9: Digestion of AaV, *A. anophagefferens*, and amplified AaV DNA by various restriction enzymes: no enzyme control (1), Hpy166II (2), XbaI (3), XhoI (4), DpnI (5), DpnII (6). DpnI and DpnII are used as negative and positive controls respectively, as they degrade either only methylated (DpnI) or unmethylated (DpnII) GATC motifs, which are expected to be unmethylated in AaV.


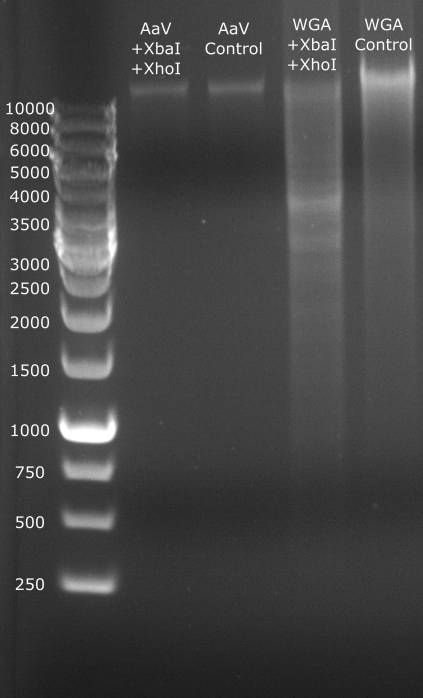


Supplemental Figure 10: Unamplified AaV DNA (lanes 2 and 3) and WGA AaV DNA (lanes 4 and 5) digested by both XbaI and XhoI enzymes together (lanes 2 and 4) or undigested (lanes 3 and 5).


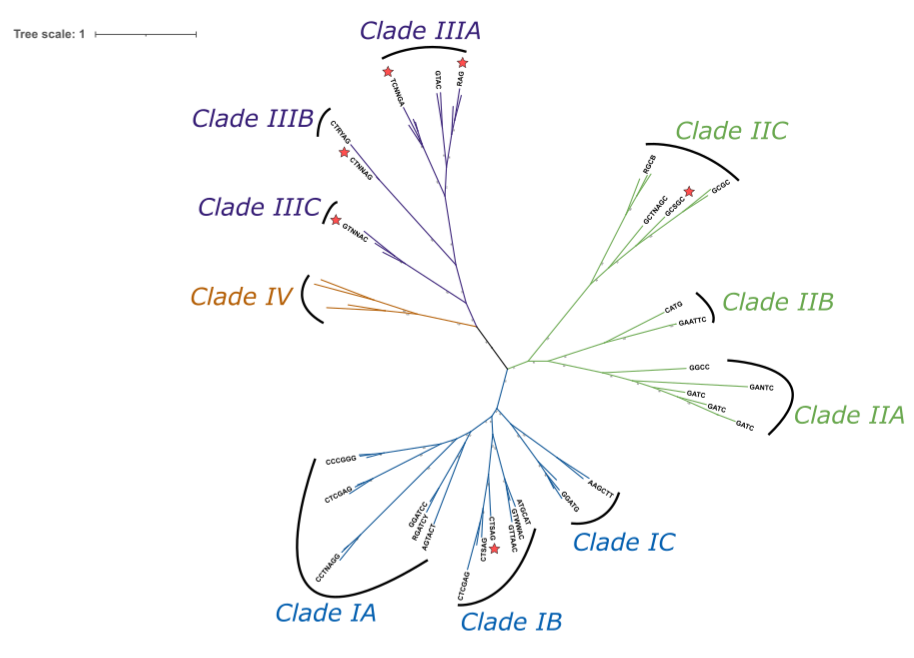


Supplemental Figure 11: Unrooted protein tree of viral type II DNA MTases. Clades are defined for clarity purposes exclusively in this study and are denoted by different colors. Stars represent the presence of an AaV-encoded methyltransferase. The colors of branches correspond with those seen in Figure 6.


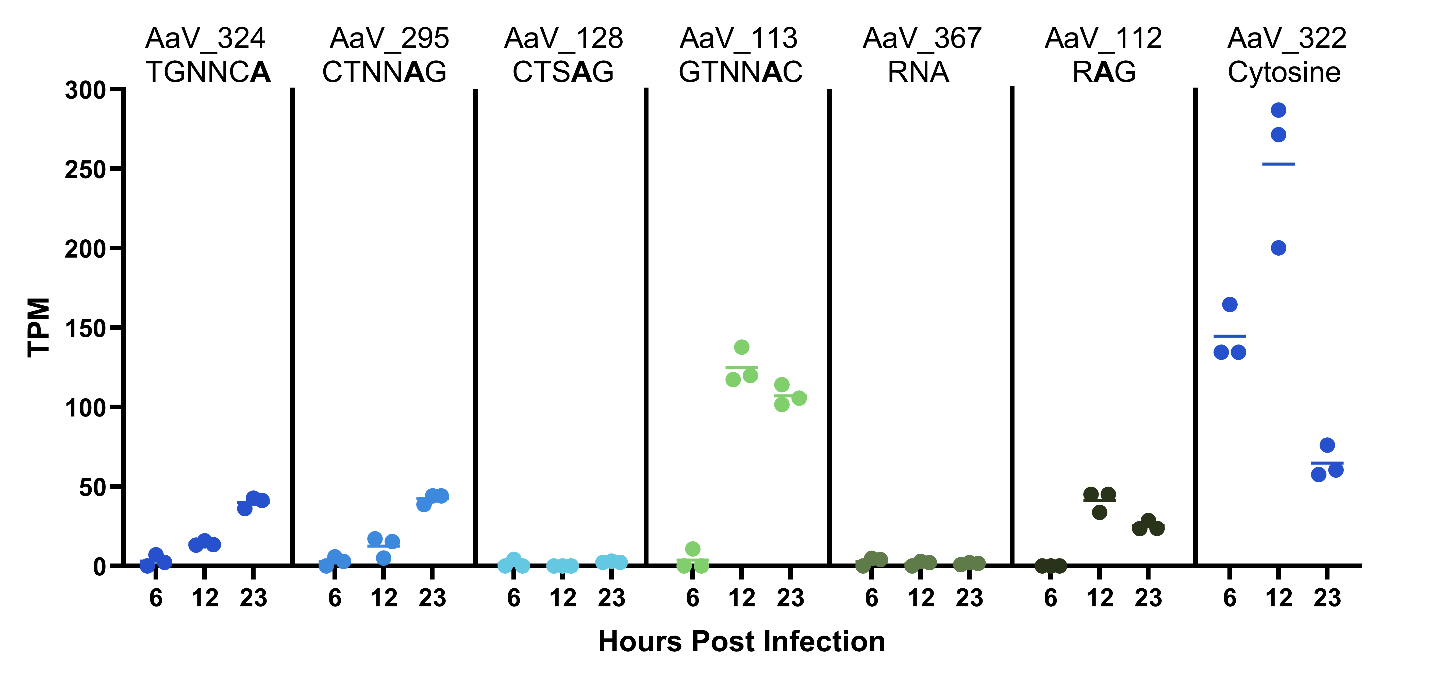


Supplemental Figure 12: Expression level of all AaV MTases represented through TPM at different stages of infection.
