# Supplementary material for: Epigenome analysis of an algae-infecting giant virus reveals a unique methylation motif catalogue
Source: PLoS One. 2025 Dec 12;20(12):e0330887. doi: 10.1371/journal.pone.0330887 (PMC12700378; doi:10.1371/journal.pone.0330887)
Supplement: S3 File — (PDF) [file pone.0330887.s003.pdf]

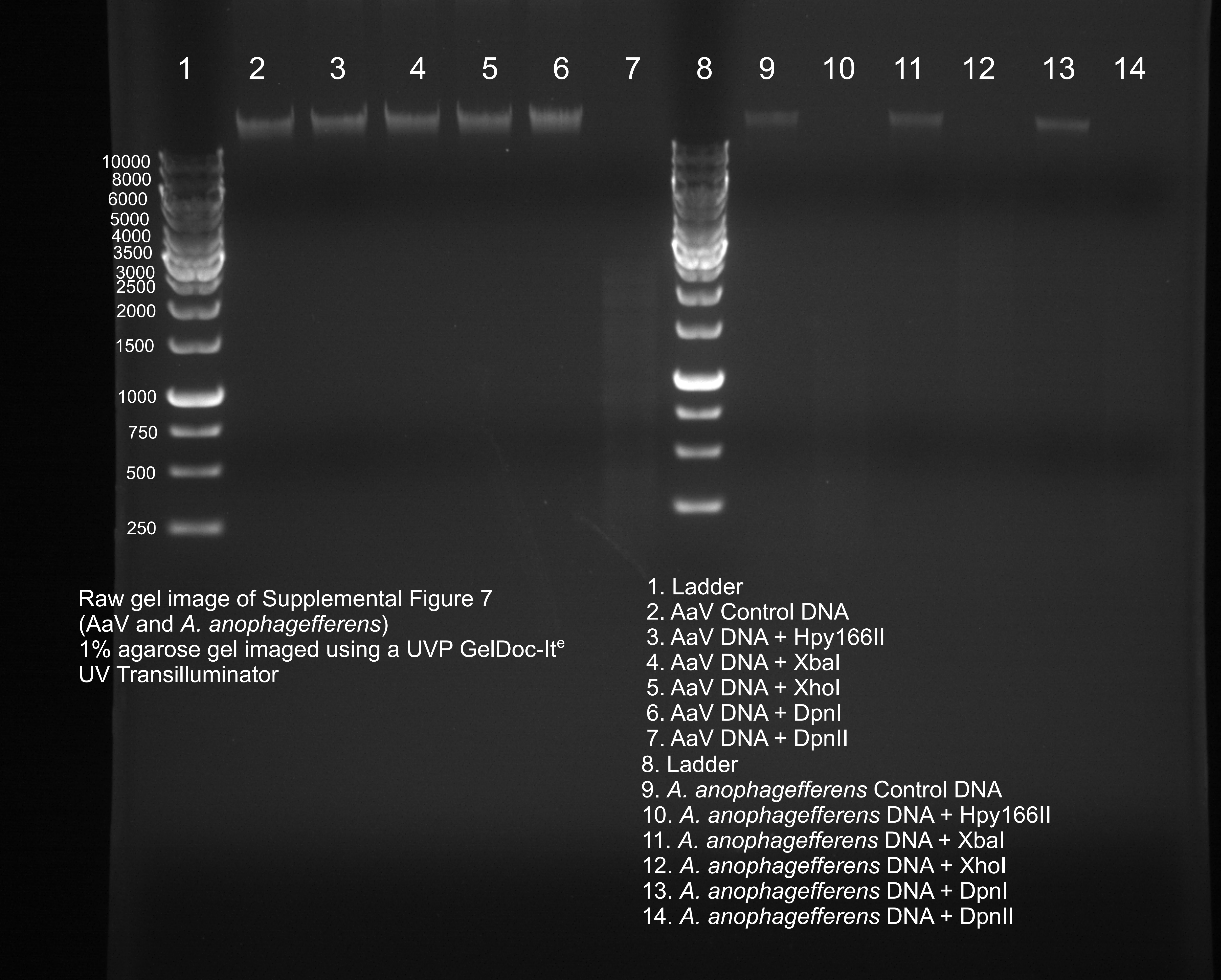

Raw gel image of Supplemental Figure 7
(AaV and *A. anophagefferens*)
1% agarose gel imaged using a UVP GelDoc-It[e]
UV Transilluminator

1. Ladder
2. AaV Control DNA
3. AaV DNA + Hpy166II
4. AaV DNA + XbaI
5. AaV DNA + XhoI
6. AaV DNA + DpnI
7. AaV DNA + DpnII
8. Ladder
9. *A. anophagefferens* Control DNA
10. *A. anophagefferens* DNA + Hpy166II
11. *A. anophagefferens* DNA + XbaI
12. *A. anophagefferens* DNA + XhoI
13. *A. anophagefferens* DNA + DpnI
14. *A. anophagefferens* DNA + DpnII

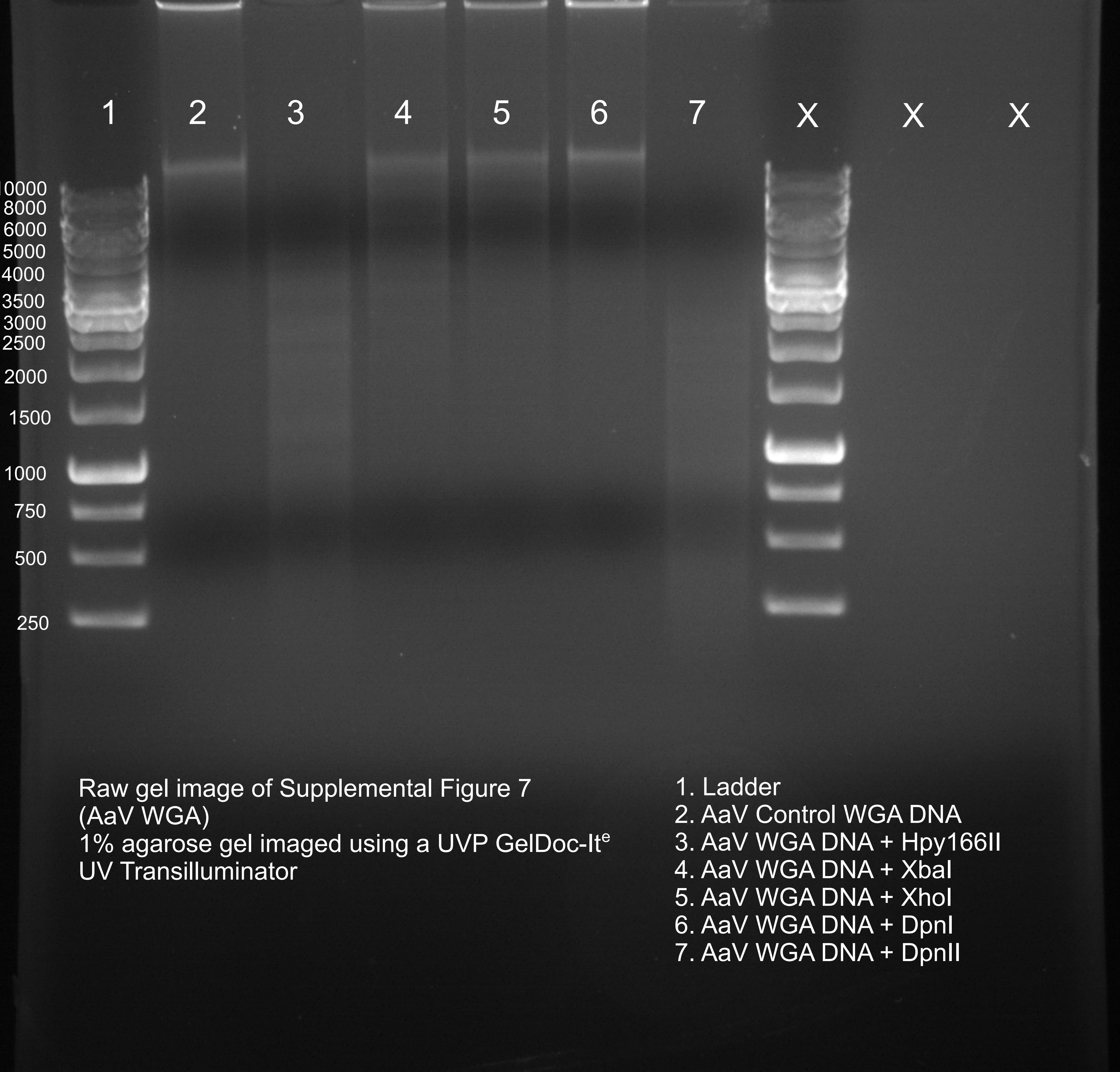

Raw gel image of Supplemental Figure 7
(AaV WGA)
1% agarose gel imaged using a UVP GelDoc-It[e]
UV Transilluminator

1. Ladder
2. AaV Control WGA DNA
3. AaV WGA DNA + Hpy166II
4. AaV WGA DNA + XbaI
5. AaV WGA DNA + XhoI
6. AaV WGA DNA + DpnI
7. AaV WGA DNA + DpnII

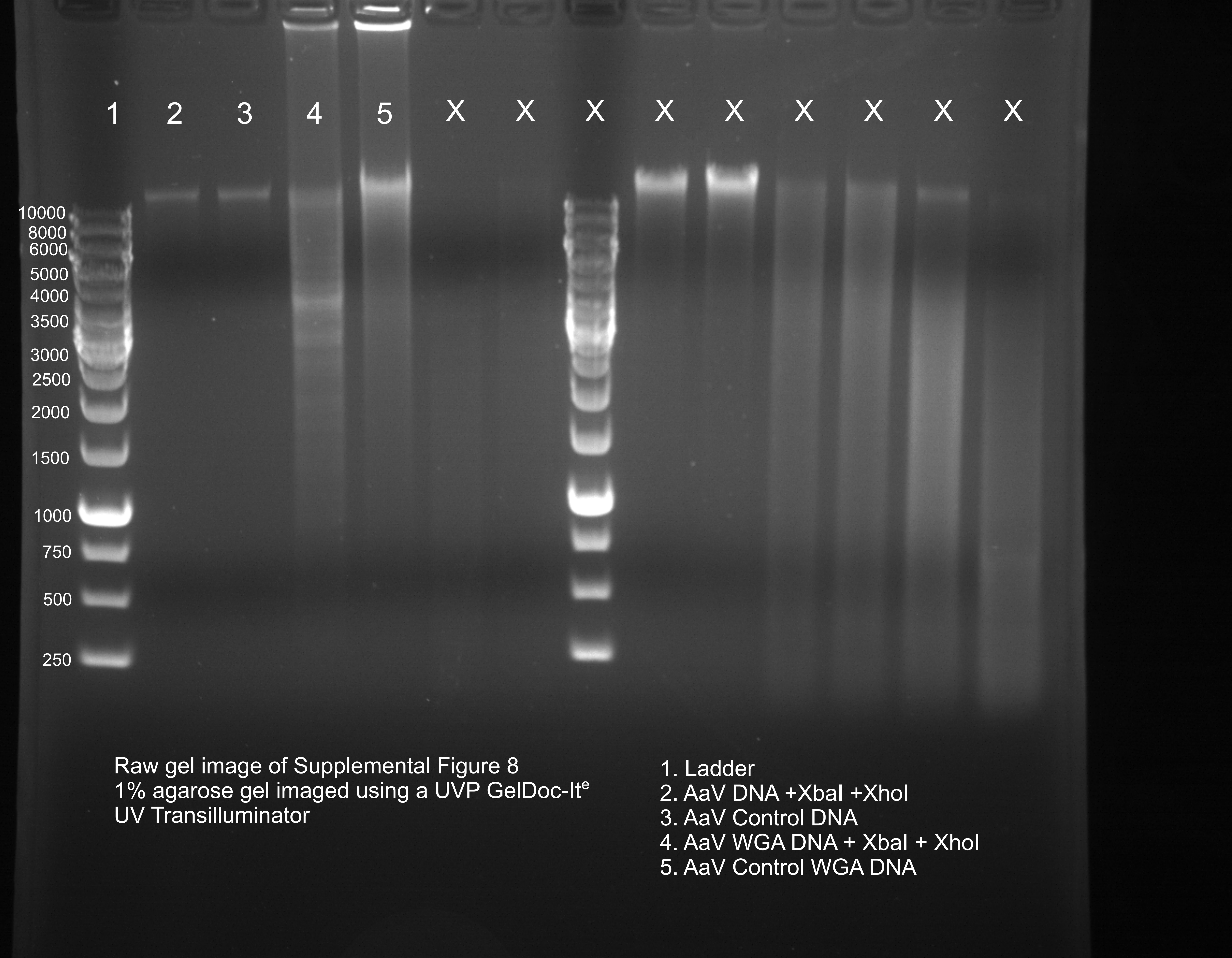

Raw gel image of Supplemental Figure 8
1% agarose gel imaged using a UVP GelDoc-It[e]
UV Transilluminator

1. Ladder
2. AaV DNA +XbaI +XhoI
3. AaV Control DNA
4. AaV WGA DNA + XbaI + XhoI
5. AaV Control WGA DNA
